# Time delay and risk of toxicity of intraosseous anaesthesia use for awake intraosseous access in children

**DOI:** 10.1007/s00063-025-01253-0

**Published:** 2025-02-27

**Authors:** Daniel Pfeiffer, Martin Olivieri, Victoria Lieftüchter, Florian Hey, Florian Hoffmann

**Affiliations:** Abteilung für pädiatrische Intensiv- und Notfallmedizin, Dr. von Haunersches Kinderspital, LMU Klinikum, LMU München, Lindwurmstraße 4, 80337 München, Germany

**Keywords:** Pediatric emergency medicine, Vascular access, Guidelines, Patient safety, Pain, Lidocaine, Pädiatrische Notfallmedizin, Gefäßzugang, Leitlinien, Patientensicherheit, Schmerz, Lidocain

## Abstract

**Introduction:**

Intraosseous access (IO) is a crucial, life-saving alternative vascular access in paediatric emergency medicine. In awake paediatric patients, the pain of drilling and flushing the marrow cavity are barriers to the use of the IO method or prompt the use of an intraosseous anaesthetic agent, which introduces the risk of dosing errors and drug toxicity. This study aims to identify the frequency of use of anaesthetic agents and analyse the time delay caused by their use.

**Methods:**

Prospective surveillance study analysing all patients, aged > 28 days to 18 years, who received one or more IO attempt(s) in and out of the hospital setting in Germany from 1 July 2017 to 30 June 2019 via the reporting mechanism of the German Paediatric Surveillance Unit (GPSU).

**Results:**

Our analysis identified 74 patients who received an IO attempt while awake. All patients were younger than 6 years old. Almost every third child (31.6%) was awake during IO use. In 18.9% of all awake patients, an intraosseous anaesthetic was used before the IO was drilled or the marrow cavity was flushed, introducing a significant time delay of approximately 3 min (*p* = 0.001) compared to IO attempts without intraosseous anaesthesia.

**Conclusions:**

Intraosseous anaesthesia prolongs the establishment of working vascular access in an emergency and introduces the risk of drug toxicity. To prevent adverse events, particular emphasis must be placed on placement without intraosseous anaesthesia, and alternative pain management (intranasal) must be considered if necessary. Training courses and guidelines should reflect the advised current practice.

## Abstract (de)

**Einleitung:**

Der intraossäre Zugang (IO) ist ein wichtiger, lebensrettender alternativer Gefäßzugang in der pädiatrischen Notfallmedizin. Bei wachen pädiatrischen Patienten stellen die Schmerzen beim Bohren und Spülen der Markhöhle Hinderungsgründe für die Anwendung des intraossären Zuganges dar oder veranlassen den Einsatz eines intraossären Anästhetikums, welches das Risiko von Fehldosierungen und Medikamententoxizität mit sich bringt. Ziel dieser Studie ist zu ermitteln, wie häufig Anästhetika eingesetzt werden, und die durch ihren Einsatz verursachte Zeitverzögerung zu analysieren.

**Methoden:**

Prospektive Beobachtungsstudie zur Analyse aller Patienten im Alter zwischen > 28 Tagen und 18 Jahren, die von 1. Juli 2017 bis 30. Juni 2019 in Deutschland einen oder mehrere IO-Anlageversuche in stationären oder ambulanten Settings erhalten haben und über das Meldesystem der Erhebungseinheit für Seltene Pädiatrische Erkrankungen in Deutschland (ESPED) gemeldet wurden.

**Ergebnisse:**

Unsere Analyse ergab 74 Patienten, bei denen ein IO-Versuch im Wachzustand durchgeführt wurde. Alle Patienten waren jünger als 6 Jahre. Fast jedes dritte Kind (31,6 %) war während der IO-Anlage wach. Bei 18,9 % aller wachen Patienten wurde vor dem Bohren des intraossären Zugangs bzw. dem Spülen der Markhöhle eine intraossäre Anästhesie durchgeführt, was im Vergleich zu IO-Versuchen ohne intraossäre Anästhesie zu einer erheblichen Zeitverzögerung von etwa 3 min führte (*p* = 0,001).

**Schlussfolgerungen:**

Eine intraossäre Anästhesie verlängert die Anlage eines funktionierenden Gefäßzugangs in einem Notfall und birgt das Risiko toxischer Medikamentennebenwirkungen. Um unerwünschte Ereignisse zu vermeiden, ist besonders darauf zu achten, dass die Anlage ohne intraossäre Anästhesie erfolgt, und bei Bedarf ist eine alternative Schmerzbehandlung (z. B. intranasal) in Betracht zu ziehen. Fortbildungsveranstaltungen und Leitlinien sollten die empfohlene aktuelle Praxis widerspiegeln.

## Introduction

Intraosseous access (IO) is a crucial, life-saving alternative vascular access in paediatric emergency medicine. The technique is reasonably safe and has high success rates of up to 98.3%. IO is the quickest vascular access method in small children when peripheral venous access is difficult to obtain, or the provider has little experience [1–4].

The pain level of drilling the needle into the bone is reported to be comparable to that of a peripheral or central venous access placement. In contrast, the pain caused by infusion (i.e. the first flush of the medullary cavity) is reported to be significantly higher. Furthermore, in recent years, the use of anaesthetic agents to reduce the perceived endostal pain caused by applied volume or drugs has been recommended in guidelines and procedure manuals. Therefore, it is understandable that some providers are worried about pain caused by intraosseous access and tend to consider some form of analgesia, be it local, systemic or intraosseous [3–8].

Due to the relatively low maximum dose of lidocaine in small children (of 5 mg/kg) with a narrow therapeutic range and the rare use in paediatric emergency medicine, the risk of dosing error is significantly high. In recent years, several adverse events due to medication errors with local anaesthetics, of which some had lethal outcomes, were observed. In Germany, this led the BfArM (German Federal Institute for Drugs and Medical Devices) to release a statement advising against intraosseous lidocaine use in paediatric IO placement [9–11].

## Materials and methods

Data were collected and analysed as described in Pfeiffer et al. and Schwindt et al. [2, 12]: In brief, our prospective surveillance study collected all patients aged > 28 days to 18 years who received one or more intraosseous access attempt(s) in and out of the hospital setting in Germany from 1 July 2017 to 30 June 2019 via the reporting mechanism of the German Paediatric Surveillance Unit (GPSU).

## Results

Our prospective nationwide surveillance study in Germany analysed 234 children, aged > 28 days to 18 years, who received one or more intraosseous access attempt(s) in the context of a GPSU survey from July 2017 to June 2019. Of those, we excluded 160 patients who received cardiopulmonary resuscitation (CPR) or were unconscious during IO access attempts. This method identified 74 cases in which the patient received intraosseous access while awake. All patients were younger than 6 years old.

Almost every third child (31.6%) is awake during intraosseous access use. In 18.9% (*n* = 14) of all awake patients, an intraosseous anaesthetic was used before the IO was drilled or the marrow cavity was flushed. In our study, this leaves close to every fifth patient at risk of potentially life-threatening complications from lidocaine dosing errors and consecutive toxicity. In all 14 patients, peripheral venous access was attempted before IO was established (see Table 1).

**Fig. 1:**
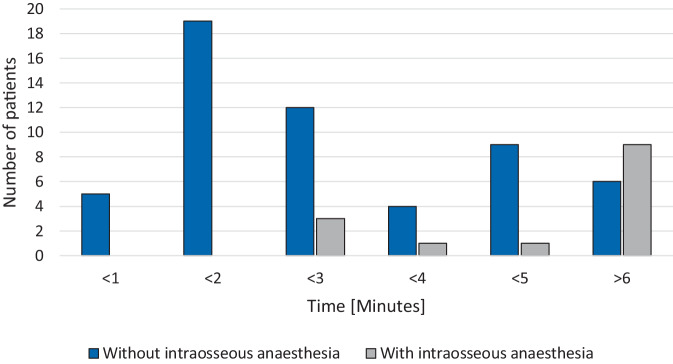
Time to successful intraosseous access in minutes with and without intraosseous anaesthesia

Furthermore, intraosseous anaesthetics led to a significant time delay (Kruskal–Wallis test *p* = 0.001). It took, on average, more than 3 min longer to establish intraosseous access when intraosseous anaesthetics were used (median < 3 min vs > 6 min) (see Fig. 1). All 14 patients were younger than 6 years and survived to discharge. In addition, only 1 out of 14 provider’s institutions had a guideline or standardised operating procedure (SOP) for IO use. In all, 71.4% of providers received some training before IO use.

## Discussion

Intraosseous anaesthesia prolongs the establishment of working vascular access in an emergency. Furthermore, intraosseous lidocaine use in small children provides an inherent risk of drug toxicity. To prevent adverse events, particular emphasis must be placed on placement without intraosseous anaesthesia, and alternative pain management (intranasal) must be considered if necessary, for example, esketamine (2 mg/kg).

Furthermore, the topic should be addressed in guidelines and training courses dealing with vascular access establishment in critically ill children with a focus on alternative pain management strategies. Standard operating procedures should reflect the advised current practice. Further research is necessary to evaluate possible changes in local anaesthesia usage rates after the BfArM statement [9, 13].

Three patients in our study cohort received an intraosseous access during a seizure/status epilepticus. Meaning they were likely unconscious at the time of puncture and did not need any form of analgesia, especially, for the pain of the application of antiepileptic drugs with small volumes. Considering all these implications of lidocaine use, we propose a practical stepwise approach: 1) If a patient is awake and the physician is concerned about the pain of placement, re-evaluate the indication for IO use—is it a life-threatening situation? Are fluids or life-saving medications only available intravenously or intraosseously necessary to stabilise the patient? 2) If IO is deemed necessary and it is a life-threatening, urgent situation (in which the consciousness of the critically ill patient is likely impaired), potential pain is secondary to the primary goal of administering life-saving therapy. If analgesia is necessary and the clinical situation of the patient allows it, alternative anaesthetic methods should be used to administer a safer form of analgesia (e.g. intranasally) before IO is attempted (two-stage approach) [13, 14].

For example, the intranasal application of esketamine (2 mg/kg) or fentanyl (1.5–2 µg/kg). There is sufficient evidence in the literature supporting the use of ketamine and fentanyl with a sound safety profile. Multiple studies report a similar pain reduction efficacy of fentanyl and ketamine when used intranasally. Prescott et al. (2023) reports similar pain relief to intramuscular morphine and intravenous fentanyl. Minor transient adverse events (e.g. dizziness, sleepiness, nausea, bad taste in mouth) occur more frequently when using subdissociative dose ketamine. No severe adverse events were reported in Reynolds et al. (2017) and Graudins et al. (2013). Intranasal ketamine causes more sedation compared to intranasal fentanyl. Intranasal subdissociative dose ketamine should be preferred when an increased sedational component is necessary, or when there are contraindications for opioids. There is insufficient literature on the direct comparison of intranasal analgesia compared to intraosseous lidocaine in paediatric patients—and, with respect to the more recent warning letters and position papers, there hopefully never will be, as the use of lidocaine as anaesthetic for intraosseous access should be considered obsolete [14–20].

## Conclusion

Intraosseous anaesthesia prolongs the establishment of working vascular access in an emergency. Furthermore, intraosseous lidocaine use in small children provides an inherent risk of drug toxicity. To prevent adverse events, particular emphasis must be placed on placement without intraosseous anaesthesia, and alternative pain management (intranasal) must be considered if necessary. Furthermore, the topic should be addressed in guidelines and training courses dealing with vascular access establishment in critically ill children. Standard operating procedures should reflect the advised current practice. Further research is necessary to evaluate possible changes in local anaesthesia usage rates after the BfArM (German Federal Institute for Drugs and Medical Devices) statement.

## Limitations

This study has multiple limitations that apply. These are similar to the limitations in Pfeiffer et al. (2023) and Schwindt et al. (2022). In short, GPSU’s voluntary reporting mechanism, using only GPSU-reporting hospitals, introduces a significant underreporting and selection bias. Our study method might miss cases from nonreporting institutions, which could mean that certain practices or adverse events are underrepresented in our cohort. Due to our stringent exclusion criteria (CPR or unconscious) and the resulting (relatively) small study cohort, the broader applicability of our findings is limited. Since the use of intraosseous access in paediatric emergencies is a very rare event, further large registry studies (potentially in cohorts with mandatory instead of voluntary reporting) are needed to systematically evaluate larger cohorts of awake intraosseous access use in children. Larger cohorts would allow a more representative assessment of the frequency of use, the time difference and better control for confounding variables. The underrepresentation of older paediatric patients (> 6 years of age) further limits the generalisability of our results, especially for those older patients, who might possess different pain tolerances or requirements for anaesthetic management. Retrospective cognitive reconstruction of events, especially relating to information about procedure times (e.g., time to first IO access), is known to be inconsistent [2, 12].

**Table 1:** Characteristics of patients with intraosseous access lidocaine use (*n* = 14)

	*n*	%
**Age**		
*1–6 years*	9	64.3
*1–12 months*	5	35.7
**Diagnosis**		
*Shock/Sepsis*	4	28.6
*Seizure/Status epilepticus*	3	21.4
*Diabetic ketoacidosis*	3	21.4
*Thermal injury*	2	14.3
*Poor peripheral venous status*	1	7.1
*Dehydration/Exsiccosis*	1	7.1
**Alternative access attempts**		
*Alternative access attempted*	14	100.0
– Peripheral venous access attempt	14	100.0
– Central venous catheter	1	7.1

## Data Availability

The authors will make the raw data supporting this article’s conclusions available without undue reservation.

## Declarations

All surveillance studies of the GPSU are based on a common study and data protection protocol. The local ethics committee of the Rhineland–Palatinate Medical Association and the state representative for data protection in Rhineland–Palatinate have approved this protocol. A waiver of written informed consent was granted by the Ethics Committee of the State Medical Association of Rhineland–Palatinate (number 2020-15400_1) and the state representative for data protection in Rhineland–Palatinate as participants were not subjected to any study-related procedures and data were collected in a completely anonymous form. All analysed data involving human participants were collected in accordance with the ethical standards outlined in the 1964 Helsinki Declaration and its later amendments or comparable ethical standards. In addition to the general ethical approval of the GPSU, the study was approved by the data protection office and the ethics committee of the medical faculty of the Ludwig-Maximilians-University, Munich No. 641-16 (19-12-2016).

The supplement containing this article is not sponsored by industry.
